# Evolution of Repetitive Elements, Their Roles in Homeostasis and Human Disease, and Potential Therapeutic Applications

**DOI:** 10.3390/biom14101250

**Published:** 2024-10-02

**Authors:** Jeffrey Snowbarger, Praveen Koganti, Charles Spruck

**Affiliations:** Cancer Genome and Epigenetics Program, Sanford Burnham Prebys Medical Discovery Institute, La Jolla, CA 92037, USA; jsnowbarger@sbpdiscovery.org (J.S.); pkoganti@sbpdiscovery.org (P.K.)

**Keywords:** repetitive elements, transposable elements, viral mimicry, epigenetic alteration, genomic instability, co-option, novel antigens

## Abstract

Repeating sequences of DNA, or repetitive elements (REs), are common features across both prokaryotic and eukaryotic genomes. Unlike many of their protein-coding counterparts, the functions of REs in host cells remained largely unknown and have often been overlooked. While there is still more to learn about their functions, REs are now recognized to play significant roles in both beneficial and pathological processes in their hosts at the cellular and organismal levels. Therefore, in this review, we discuss the various types of REs and review what is known about their evolution. In addition, we aim to classify general mechanisms by which REs promote processes that are variously beneficial and harmful to host cells/organisms. Finally, we address the emerging role of REs in cancer, aging, and neurological disorders and provide insights into how RE modulation could provide new therapeutic benefits for these specific conditions.

## 1. Introduction

Not long after experiments which implicated DNA as the genetic material were published in 1944 [1], independent groups made two other important findings that helped inform our current understanding of the genome and the nature of REs. The first of these was McClintock’s discovery of “controlling elements” (which we now understand to be transposons) in maize, capable of inserting themselves elsewhere in the host genome and altering phenotypes [2]. Second, in DNA reannealing experiments using multiple organisms, several groups found that large portions of DNA reassociated much faster than would be expected if the DNA of these organisms was largely comprised of unique nucleotide sequences. This finding suggested that the DNA of these organisms contained many repetitious sequences [3,4]. These early experiments highlighted the characteristics of REs that we now recognize as crucial for their (dys)regulation of cellular and organismal functions: they are indeed repetitive, and certain elements can be mobilized throughout the genome.

REs are found in the genomes (and organelles [5,6,7]) of organisms across the phylogenetic spectrum—from prokaryotes and other single-celled eukaryotes all the way to humans [8,9,10,11,12,13]. Indeed, prokaryotic clustered regularly interspaced short palindromic repeats (CRISPRs), which have garnered much interest as a genome editing tool, are REs that provide the simplest living organisms with a sort of adaptive immune system [14,15]. REs comprise at least 50% of the human genome and are made up of various classes, which are described in subsequent sections [13,16,17,18]. Until recently, REs and other non-coding sequences were thought to simply be “junk” DNA. However, the discoveries that a majority of the genome is transcribed and that non-coding RNAs (ncRNAs) have several different functions have largely reversed the “junk” DNA viewpoint [19]. Furthermore, advances in DNA sequencing technologies have increased the ability to sequence repetitive regions that were omitted in initial sequences of the reference human genome. This advance in sequencing capability will continue to enable new insights into RE characteristics and their influences on host genomes [18]. A significant body of work has now been published that suggests REs are, in fact, key players that can directly or indirectly regulate a variety of cellular and organismal processes. Therefore, in this review, we classify REs and the general ways they contribute to both beneficial and pathological processes and explore the roles of REs in specific disorders and the contexts in which RE modulation could provide therapeutic benefits for these disorders.

## 2. Classification and Evolution of REs

At the broadest level, REs can be grouped by their dispersion patterns into two different classes. There are tandem repeats, whose repetitive units are arrayed head to tail next to each other, and interspersed repeats, whose repetitive units are dispersed at different locations throughout the genome. Tandem repeats comprise various types of satellite repeats. Also, while not the subject of this review, one could consider the genes encoding ribosomal RNA as tandem repeats because they are tandemly arrayed in many repeating copies to support the high level of protein synthesis needed in cells [20]. Interspersed REs encompass the two types of transposable elements (TEs): RNA and DNA transposons. RNA transposons are also called type or class 1, whereas DNA transposons are type or class 2 [16]. To be comprehensive, we classify both tandem and interspersed repeats below; however, later sections regarding the roles of REs in disease and therapeutic applications of RE modulation are more heavily focused on the interspersed repeats. The general classifications outlined in this text can be viewed in Supplementary Figure S1. Additionally, several excellent papers that detail both the classification and general structures of REs are provided in references [16,21,22,23,24,25,26,27,28,29].

### 2.1. Tandem Repeat Classification and Evolution

The various satellite repeats comprise ~8–10% of the human genome [30]. Satellite repeats are found largely in (peri)centromeric and (sub)telomeric regions and help make up the constitutive heterochromatin compartment [16,21,31]. Satellite repeat subclasses differ in their chromosomal localization, the length/sequence of their repetitive monomers, and the total size of their arrays. Briefly, microsatellites (also called short tandem repeats; STRs) and minisatellites (also called variable number tandem repeats; VNTRs) are tandem repeats whose repetitive units are <10 and 10–100 base pairs (bp), respectively. Perhaps the most famous of these are the telomeric satellite repeats [32]. In addition to micro and minisatellites, other major players are the α-satellite repeat and human satellites I–III (Sat I–III). The α-satellite repeat is the primary centromeric repeat and is found in higher-order repeat arrays that can span several megabases in all centromeres [30,33]. Following the α-satellite family, Sat I–III are the next largest subclass of satellites and are the sequences whose discovery coined the term “satellite” DNA—which has now been broadened to encompass tandem repeats of varying sizes. Sat I–III have different chromosomal distributions and motif sequences (reviewed in [21]).

Satellite DNA sequences are some of the most rapidly evolving elements of the genome and may even contribute to reproductive barriers resulting in speciation [34]. A large driver of this rapid evolution is the inherent instability of tandem repeat sequences through a variety of genomic DNA processes. For example, unequal crossing over during homologous repair (HR) of double-stranded breaks (DSBs) is a process that can lead to copy-number changes in satellite sequences, and if this occurs in the germline these changed proportions can be passed down [21,35]. Ectopic crossovers can also occur between repeat sequences on completely different chromosomes, resulting in translocations [21,22]. Copy-number changes can also occur through multiple mechanisms of improper replication [31,36,37]. Unequal crossing over and other improper replication events contribute to the two main models of satellite DNA evolution: the library model and concerted evolution by molecular drive. Aspects of both models may reflect the reality of satellite DNA evolution [38]. The library model states that closely related species contain conserved sets of satellite families from a common ancestor, but each family is amplified to different degrees in each species. Concerted evolution suggests that mutations to existing satellite DNA monomers can be spread across or removed from whole arrays (homogenization) through mechanisms like unequal crossing over and TE activity. Through sexual reproduction, newly homogenized satellite arrays are fixed in subsequent offspring, eventually resulting in species-specific satellite arrays [34,39]. A separate mechanism of centromeric satellite DNA evolution is called centromere (or meiotic) drive. This is a phenomenon where centromeric satellites that enable better spindle binding during asymmetric female meiosis are preferentially drawn to the egg pole and not lost in polar bodies [40]. 

### 2.2. Interspersed Repeat Classification

TEs comprise at least 40% of the human genome and are often called parasitic or selfish elements, as they propagate throughout host genomes in attempts to make more copies of themselves. As mentioned above, the broadest way to characterize TEs is through the intermediate by which they reintegrate into the genome. RNA transposons (or retrotransposons) do so through an RNA intermediate that is reverse transcribed back into DNA for integration. Conversely, DNA transposons (or transposons) have their genomic DNA sequence excised, and this sequence is then reintegrated into the genome. Retrotransposon transposition is replicative in nature and can be thought of as a “copy and paste” mechanism, whereas DNA transposon transposition is non-replicative and can be thought of as “cut and paste”.

Retrotransposons can be further subdivided by their mechanisms of reintegration into long terminal repeat (LTR; utilizing an integrase), target-primed non-LTR, or tyrosine recombinase classes [23]. The LTR class (~8% of the genome) is composed of endogenous retroviruses (ERVs) whose presence in the genome is likely to have been the result of retroviral infection in germline cells of human evolutionary ancestors [41]. While many ERV families are largely present as fragments throughout the genome due to mutations over evolutionary time [42], the full-length sequence structure of the active HML-2 subfamily of human ERV-K (HERV-K) encodes the retroviral gag, pro, env, and pol genes sandwiched between 5′ and 3′ LTRs [16,41]. Reverse transcription utilizes tRNAs as primers, and after reverse transcription, the cDNA is reintegrated into the genome via an integrase encoded by the pol gene. Notably, 5′ and 3′ LTRs of ERVs often undergo homologous recombination, resulting in the deletion of all internal sequences and a composite solitary LTR (or “solo LTR”) that has unique portions of both the 3′ and 5′ LTRs [43,44]. These solo LTRs are ubiquitous with around 577,000 annotations within the human genome and can have regulatory activity on nearby genes [44,45]. For HERV-K, the active HML-2 subfamily contains about 89 proviruses and 1200 solo LTRs [46].

Non-LTR retrotransposons include the long interspersed nuclear elements (LINEs) and short interspersed nuclear elements (SINEs). The main, autonomous (capable of their own retrotransposition) non-LTR retrotransposon in humans is the LINE-1 element. Full-length LINE-1 contains two open reading frames (ORFs) that encode a 40 kDa RNA-binding protein (ORF1p) and a 150 kDa enzyme that has both endonuclease and reverse transcriptase activity (ORF2p) [47]. These ORFs are flanked by 5′ and 3′ untranslated regions (UTRs) and a 3′ polyadenylation signal (pAS) and poly(A) tract. Interestingly, a third, primate-specific ORF has been described within the 5′ UTR of LINE-1. This ORF, coined ORF0, is an antisense ORF that can be transcribed and translated. ORF0 gene products can enhance LINE-1 mobility [48]. LINE-1 utilizes what is called target-primed reverse transcription to reintegrate into the genome, and notably, reverse transcription often ends early in this mechanism, resulting in 5′ truncated LINE-1s. Because the 5′ UTR of LINE-1 contains a POLII promoter, 5′ truncated LINE-1s do not undergo further transposition events. It is important to note that despite constituting such a large proportion of the human genome (~21%), there are only about 100 retrotransposition-competent LINE-1 sequences due to 5′ truncation and other mutations within internal sequences. Thus, the bulk of that 21% of the genome represents truncated and fragmented ancient relics of past transposition activity [28,49]. SINEs rely on the LINE-1 machinery for their transposition and have evolved similar features (like a poly(A) region) to LINE-1 [23]. SINEs, like the ALU element, are thought to have originated from noncoding genes like tRNA, 7SL, and 5S. These noncoding genes are all transcribed by POLIII, and accordingly, ALU, which comprises about 12% of the genome, is also transcribed by POLIII. Not fully a SINE, the SVA element, which comprises < 1% of the genome, is a non-autonomous element composed of SINE, VNTR, and ALU elements. SVA is transcribed by POLII and also mobilized by LINE-1 machinery [23,27]. Tyrosine recombinase (YR) retrotransposons have similar structures to LTR retrotransposons, but instead of the integrase, they encode a YR for reintegration. The presence and activity of YR DNA retrotransposons has been described in other eukaryotic species (i.e., DIRS1 [50]). There do not appear to be recognizable YR retrotransposon sequences in the human genome; however, there are two genes that encode proteins containing DIRS-derived domains [25,51].

DNA transposons can also be classified by their mechanism of reintegration into the genome. Briefly, the four main classes are the DDE (named for the catalytic amino acids in their recombinase enzyme), YR, rolling circle, and self-synthesizing transposons. Consensus structures and information on the mechanisms of transposition can be found in [23,52]. Collectively, DNA transposons comprise about 3% of the human genome, and members of the hAT and Mariner/Tc1 superfamilies comprise a majority of that 3% [25,53]. Despite comprising a significant proportion of the human genome, DNA transposons are considered “fossils” as there are no members actively capable of transposition [25,54]. As “cut and paste” TEs, their transposition events are not replicative; however, it is thought that DNA transposons have accumulated many copies of their sequences through transposition to yet-unreplicated sites during DNA replication [55,56] and by homologous recombination repair of the DSB that remains at the excision site. The latter method can lead to deletions within the original transposon sequence, rendering the repaired sequences non-autonomous (examples of this are the families of miniature inverted-repeat transposable elements; MITEs) [57,58,59].

#### Interspersed Repeat Evolution

Despite the pervasive presence of TEs across the phylogenetic spectrum, pinning down evolutionary origins and relationships for each family and their subfamilies is a daunting task [60]. Exploring these evolutionary relationships between TEs is mainly achieved through a phylogenomic framework, integrating taxonomic distribution of the elements and phylogenetic analyses of their shared proteins. Aside from SINEs and ERVs, which probably originated from noncoding genes and exogenous retroviruses, respectively, determining the evolutionary origins and relationships of other TEs can be confounded by two main factors [23]. The first is that a majority of TEs probably underwent periods in their evolutionary histories where, in addition to vertical transmission, they were also capable of horizontal transmission (analogous to infection) and thus integrated their sequences into the genomes of other organisms. This horizontal transmission could even have taken place between evolutionarily distant species, convoluting the picture on the origins and history of TEs [61]. Second, TEs can be chimeric through “modular evolution,” meaning that certain subfamilies of TEs utilize genes from two different superfamilies. For example, YR retrotransposons show sequence homology to LTR retrotransposons in their reverse transcriptase domains, but the YR domain seems to be closer to the YR domains of cryptons—a DNA transposon subfamily. Another example of this modular evolution or chimerism of TEs is found in the integrase structure between LTR retrotransposons and various DNA transposons; all utilize a DDE integrase. Despite these confounding factors, the conservation of a motif known as the RNA recognition motif (RRM) in several enzymes crucial for TE integration suggests a pre-eukaryotic emergence of the core transposition machinery [23,60,62]. While the evolution of TEs themselves is complex and convoluted, the ability of TEs to drive evolution of their host organisms is clearer. In addition to transposition, TEs undergo many of the same recombinational DNA processes mentioned above for the evolution of satellite repeats. As expanded upon below, after transposition or recombinational events, TEs can rapidly alter the evolutionary trajectory of their hosts due to the fact that they harbor intrinsic regulatory sequences. In other words, upon transposition or some other genomic rearrangement involving homologous TE sequences, TEs can provide new regulatory functions to neighboring genes. Interestingly, there are also cases of convergent evolution where the same gene in different organisms acquires regulation by different TEs [63].

## 3. RE Regulation and the Roles of REs in Both Beneficial and Pathological Processes

### 3.1. General Methods of RE Regulation

Interestingly, escape from the silencing mechanisms that normally repress RE activity can result in both beneficial and harmful activities, which are described subsequently. That said, it is important to mention how REs are regulated prior to discussing their roles within hosts. While there are examples of coordinated RE activity in germ cells, embryonic development, and differentiated somatic cells, REs are typically silenced through a variety of epigenetic and RNA-based mechanisms to prevent their potentially deleterious effects to the host genome [26]. Epigenetic mechanisms include DNA methylation, histone modifications, and a plethora of zinc-finger proteins that help recruit epigenetic “writer” enzymes to different REs [28,64]. DNA methyltransferases (DNMTs) establish (DNMT3a/b) and maintain (DNMT1) DNA methylation patterns, which are essential for RE silencing [65]. Additionally, there are several histone methyltransferase (HMT) writers that aid in silencing REs through deposition of different repressive histone modifications. Briefly, some of the main repressive histone marks are H3K9me3 (deposited by SUV39H1/2, SETDB1, EHMT2), H3K27me3 (deposited by EZH1/2), and H4K20me1/3 (deposited by PR-SET7, SUV420H1/2, SMYD5) [66,67]. Some of these modifications may even compensate for the loss of DNA methylation in repressing REs [68,69].

**Figure 1:**
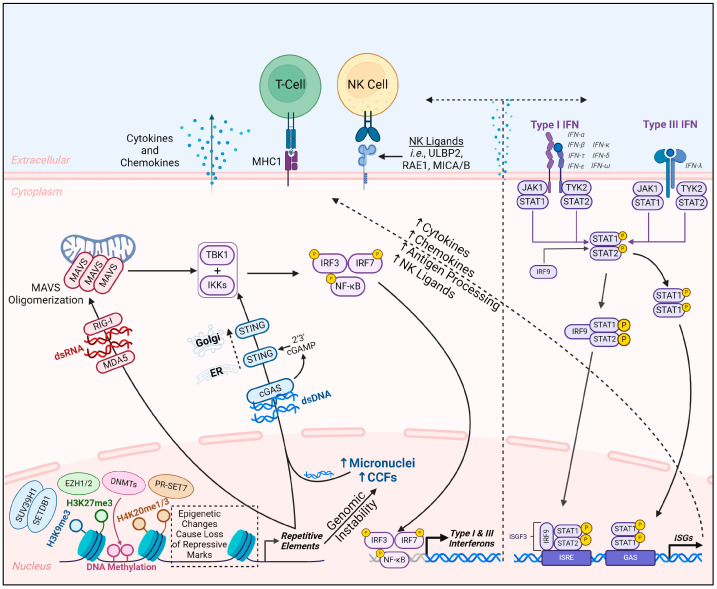
**RE derepression leads to viral mimicry and inflammation through cytosolic nucleic acid sensing pathways.** Loss of repressive epigenetic marks can derepress REs, leading to the production of cytosolic nucleic acids through retrotransposition intermediates or genomic stress (i.e., micronuclei or cytoplasmic chromatin fragments (CCFs)). Upon detection by pattern recognition receptors RIG-1, MDA5, and cGAS, downstream interferon and interferon-stimulated genes (ISGs) are expressed, leading to an inflammatory phenotype. RNA sensors activate MAVS, which oligomerizes on mitochondria, and cGAS activates STING through 2′3′ cGAMP, stimulating its translocation from the ER to the Golgi. Both pathways converge on TBK1/IKKs to phosphorylate IRF3/7 and NF-κB to induce transcription of interferons (IFNs) that, in an autocrine manner, initiate expression of ISGs. ISG expression can induce further cytokine and chemokine production, increased antigen processing, and NK cell ligand expression. This figure was inspired by and aims to expand on Figure 2 of [69] where the cytosolic dsRNA sensing pathway is explored. Other references for this figure include [70,71,72,73,74]. Created with BioRender.com (Accessed on 14 May 2024).

In mentioning the epigenetic mechanisms responsible for RE silencing, it is also appropriate to introduce the concept of “viral mimicry” and cytosolic nucleic acid sensing pathways, as these are key mediators of both beneficial and pathological inflammatory processes. As depicted in Figure 1, loss of epigenetic silencing mechanisms can result in the activation (or derepression) of retroelements, and through concomitant genomic stress or retrotransposition intermediates, cytosolic double stranded (ds)RNA and dsDNA can be produced. These cytosolic nucleic acids are then detected by pattern recognition receptors (PRRs; which normally function to detect viral nucleic acids) that stimulate inflammatory, antiviral responses through the mitochondrial antiviral signaling (MAVS) and stimulator of interferon genes (STING) proteins—hence the name “viral mimicry” [69,70]. PRRs include RIG-1, MDA5 (which both detect cytosolic dsRNA), and cGAS (a major cytosolic dsDNA sensor), and nucleic acid sensing by these enzymes resulting from retroelement derepression is implicated in both beneficial and pathological inflammation [70,71,72,73,74].

In addition to the epigenetic regulatory mechanisms described above, RNA-based mechanisms include the PIWI pathway, siRNA, and satellite-produced ncRNAs that recruit enzymes which deposit repressive epigenetic modifications [75,76]. In animals, the PIWI pathway is mainly active in the germline, and other mechanisms are active to different degrees in embryonic, germline, and somatic cells (reviewed in [16,24]). In addition, REs can also be silenced by proteins like the cytidine deaminase APOBEC3, which deaminates transiently exposed single-stranded DNA—a feature of LINE-1 reintegration into the genome [77].

**Figure 2:**
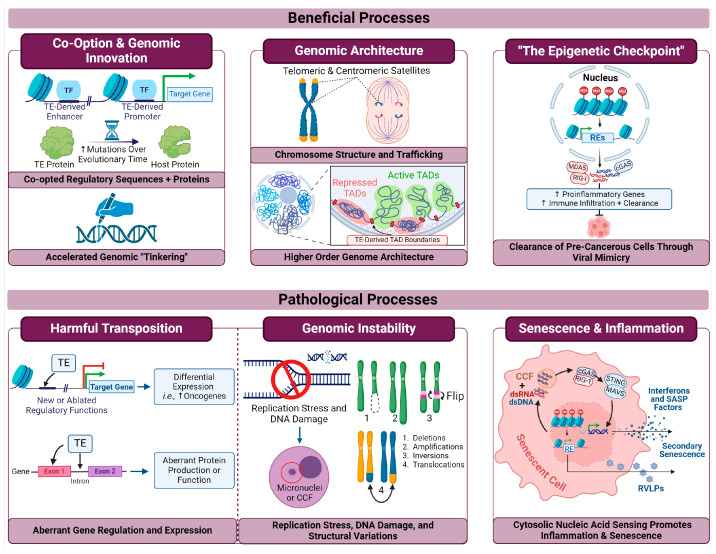
**Roles of REs in beneficial and pathological processes.** This figure describes the general methods by which RE activity in the genome can be either beneficial or harmful to the host cell and/or organism and represents what is discussed in Section 3.2 and Section 3.3. The dashed line separating the transposition and genomic instability categories signifies that transposition is also a form of genomic instability. SASP: senescence-associated secretory phenotype; RVLP: retrovirus-like particle. Created with BioRender.com (Accessed on 14 May 2024).

Both the beneficial and harmful effects of REs described subsequently occur when the silencing mechanisms mentioned above are compromised. In this section, we characterize the methods by which REs can both benefit and harm their hosts into three general categories each. This is graphically depicted in Figure 2, below.

### 3.2. Beneficial Activities of REs

The first method by which REs can promote homeostatic processes is through the addition of new regulatory circuits or functional proteins via co-option (also called exaptation or domestication). This is the process by which RE sequences or RE-derived proteins acquire new functions (immediately or latently) through transposition to a new location or mutations that confer increased fitness to the organism (Figure 2) [26,78]. Crucial to co-option is the fact that many TEs have intrinsic regulatory regions within their sequences (e.g., POLII and POLIII promoters, enhancer sequences, and splice donor sites). These intrinsic sequences enable a variety of new regulatory outcomes after transposition events, such as acting as new promoter or enhancer elements or transcription factor binding sites, or promoting transcription of fusion and/or alternatively spliced transcripts [27,79]. Examples of this co-option can be found in various biological processes such as embryonic development [27] and within the immune system [80,81]. Illustrating this point even further, chromatin immunoprecipitation with sequencing (ChIP-seq) studies have revealed that TEs contribute many binding sites throughout the genome for a variety of important transcription factors [82,83]. Proteins encoded by TEs can also be repurposed for new cellular functions. For example, the RAG1 and RAG2 proteins, crucial for V(D)J recombination to generate the diverse repertoire of B and T-cell receptors, are derived from an ancient DNA transposon called the transib transposon [84,85]. In addition, there are the DIRS-derived proteins mentioned above [51], the centromere protein B (CENP-B) [86], telomerase [87], and a variety of transcription factors [88]. Furthermore, various studies have shown that TEs imbue different ncRNA types with regulatory capabilities (discussed in [26,27,89]). One example of this is in embryonic development, where Durruthy et al. showed that certain TE-derived long ncRNAs help cells maintain pluripotency and establish the inner cell mass [90]. TE co-option and other dynamic processes of REs (like the instability of satellite sequences) provide the genome with constant innovation potential as RE activities can change existing regulatory networks, add new ones, or provide new protein functions. In other words, they have the capability to “tinker” with the genome (Figure 2). That said, we are mainly assuming that RE-driven co-option events occur in the germline or during early embryonic development as these are periods of vast epigenetic reprogramming, and the cells through which neutral or positive RE-induced genome changes would be passed on to all differentiated somatic cells and offspring.

Another beneficial function that REs impart on their hosts is the maintenance of genomic structure and architecture (Figure 2). Perhaps the most quintessential examples of this function are found through the α-satellite and microsatellite repeats of centromeric and telomeric regions that support proper chromosome trafficking during cell division and the protection of chromosome ends during DNA replication, respectively. Another appreciated role of REs regarding organization of the host genome is as binding sites for key regulators of higher-order genome structure, like CTCF. For example, Choudhary et al. showed that numerous loop anchors and topologically associated domain (TAD) boundaries are contributed by TEs, with some even being lineage-specific. These results could also be considered another example of beneficial co-option [91,92].

Finally, an unintuitive role that REs can play in their host organisms is that of an “epigenetic checkpoint” through viral mimicry (Figure 1 and Figure 2). Activation of REs can cause a variety of mutagenic effects (e.g., transposition) that can promote cancer progression. Eloquently described by Chen et al., it has been proposed that cells with the potential to become cancerous due to aberrant retroelement activation are eradicated through the inflammatory signaling of the viral mimicry process. Thus, the immune eradication of the afflicted cells results in the destruction of cell populations with altered heterochromatin that had the potential to become cancerous within the organism [69].

### 3.3. Harmful RE Activities

Paradoxically, the activity of REs that can drive co-option and genetic innovation can also cause genomic instability (Figure 2). The repetitive nature of tandem and interspersed REs gives them an inherent propensity to be involved in a variety of recombinational events based on similar homology [93]. These recombinational events can lead to deletions, inversions, translocations, and other types of DNA damage. In addition to hampering the structural integrity of the genome, these events can alter or ablate regulatory networks, leading to oncogenesis [94] or cell death. Furthermore, repetitive STR sequences are prone to expansion and contraction due to a variety of improper replication events. STR expansions have been shown to cause dozens of human diseases [95]. Another role REs play in genomic instability can be described as epigenetic remodeling. Satellite sequences themselves and transcripts of both satellite sequences and LINE-1 retrotransposons have been shown to affect the nuclear distribution and function of enzymes that deposit repressive epigenetic modifications to heterochromatic regions [96,97]. It is important to note that for both these epigenetic remodeling cases, the epigenetic landscape was already altered to begin with as the model systems used were cancer cells and a cell line model of an early-aging disease. Whether or not RE-induced epigenetic remodeling is an initiatory step of pathogenesis, these studies show it can be a subsequent step that exacerbates epigenetic alterations and can promote further harmful RE activation through loss of repressive heterochromatin.

While TE transposition can also be considered a form of genomic instability, it deserves a category of its own in terms of the methods by which REs can harm their hosts (Figure 2). TE transposition events can have a variety of effects according to the location of the newly inserted element. For example, TE insertion into genes can result in altered or deleted protein products through insertion into an exon or intron. Exon insertion can lead to chimeric proteins with aberrant functions, or nonsense or missense mutations. Intronic insertion can lead to alternative splicing or exon skipping by the introduction of new polyadenylation signal motifs, the introduction of new splice donor sites, or the disruption of canonical splicing sites [98,99,100,101,102]. Insertion elsewhere can lead to differential regulation of neighboring genes, as the newly integrated elements provide new enhancer or promoter functions or ablate the cis regulatory elements that were present. In the context of cancer, this differential regulation is particularly important for tumor suppressors and oncogenes. In addition, the epigenetic modifications associated with TEs can also differentially regulate genes near their sites of insertion [103].

The implication of various retroelements with inflammation and senescence represents another general role that REs can play in host pathology (Figure 2). In contrast to the RE-driven inflammation that enables the eradication of potentially cancerous cell populations, retroelement activation can also promote chronic low-level inflammation (termed sterile inflammation) that is harmful to the host organism [104]. Senescence, in addition to the characteristic cell cycle arrest due to several factors (e.g., DNA-damage-induced, oncogene-induced), can contribute to sterile inflammation through the senescence-associated secretory phenotype (SASP) [105]. In recent years, several studies have shown that RE detection by PRRs in the cytosol plays a causal role in promoting senescence and the SASP. For example, senescent cells produce cytoplasmic chromatin fragments (CCFs) that stain for DNA damage and heterochromatin markers, and these CCFs promote SASP through the cGAS-STING pathway [106]. In addition, LINE-1 transcription has been shown to be elevated during senescence and promote type 1 interferon inflammatory responses [107]. Moreover, it has been shown that HERV-K-derived cDNA also promotes inflammation in senescent cells through the cGAS-STING pathway. HERV-K transcription also led to the production and secretion of retrovirus-like particles (RVLPs). Strikingly, conditioned media containing RVLPs from senescent cells induced senescence and promoted inflammation in non-senescent cells of the same type [108]—a process termed secondary senescence [109].

## 4. Derepression of REs in Cancer, Aging, and Neurological Disorders

The derepression of REs, particularly the subsequent transcription and mobility of TEs across the genome, can potentially cause deleterious mutations, gene disruptions, and chromosomal rearrangements, as discussed above. These harmful events can potentially lead to the initiation and progression of several human diseases. In this section, we summarize and discuss in detail the potential roles of REs in the origin and development of human diseases, with a particular focus on cancer.

### 4.1. Implication of REs in Carcinogenesis

Altered expression of REs is emerging as a hallmark of cancer cells [110,111,112]. However, whether RE activity is a cause or consequence of cancer development remains a complex question. Aberrant expression of REs has been implicated in the development of several human malignancies, including breast cancer [113], prostate cancer [114], melanoma [115], leukemia [116], and glioblastoma [117]. In a cancer context, it is assumed that repressive regulatory mechanisms are altered or compromised, leading to RE derepression. Derepressed REs can silence tumor suppressor genes, activate oncogenes, and induce genomic instability. In this section, we discuss how REs can potentially contribute to the initiation and development of human cancers.

#### 4.1.1. TEs Can Inactivate Tumor Suppressor Genes

Derepressed TEs can randomly integrate elsewhere in the genome, thereby leading to insertional mutagenesis. LINE-1 and ALU sequences are common culprits thought to induce insertional mutagenesis [110]. Several studies have reported the reactivation of LINE-1 in several human cancers due to loss of DNA methylation [118,119,120]. Additionally, Rodic et al. showed that several human cancers exhibited LINE-1 expression at the transcriptional and protein levels, whereas its expression in normal somatic cells was absent [121]. Miki et al. showed disruption of the APC tumor suppressor gene in a human colon cancer patient via exonic LINE-1 insertion [122]. In lung squamous cell carcinoma, exonic LINE-1 insertion was shown to inactivate the tumor suppressor FGGY [123]. These studies indicate that when LINE-1 is expressed, it can potentially integrate into tumor suppressors and act as a mutagen through retrotransposition.

Several studies have also implicated ALU sequences in the disruption of tumor suppressor genes. For example, recombination events between ALU repeats have been implicated in generating deletions and rearrangements of the tumor suppressors P53 and VHL [124,125]. Several genes associated with cancer that are modulated via insertion of TEs, including LINE-1, ALU, and LTR retrotransposons, have been reviewed elsewhere [126]. Collectively, these studies indicate that TE activation can disrupt normal cellular functions through the inactivation of tumor suppressor genes and contribute to cancer initiation and progression.

#### 4.1.2. TEs Can Activate Oncogenes

In addition to inactivating tumor suppressors, derepressed TEs can serve as promoters or enhancers that drive the expression of oncogenes—a process termed onco-exaptation [127]. A quintessential example of this was reported by Morse et al., where the intronic insertion of LINE-1 into the MYC oncogene drove breast ductal adenocarcinoma [128]. Additionally, LINE-1 insertion was shown to promote the activation of the MET oncogene [129]. Furthermore, studies examining Hodgkin’s lymphoma, breast cancer, and acute myeloid leukemia (AML) have reported that retrotransposons can serve as transcription factor binding sites, promoters, or enhancers for oncogenes [130,131,132,133,134]. These studies highlight that the derepression of TEs could be a driver of cancer through the activation of oncogenes. Moreover, a study performed on several cancer types demonstrated that onco-exaptation is a widespread event that occurs in many cancers [135]. Further studies should elucidate whether onco-exaptation and the mechanisms enabling it occur in a cancer-specific manner.

#### 4.1.3. REs and Their Role in DNA Damage and Genomic Instability

Several studies have indicated that RE activation can cause DNA damage and genomic instability in a variety of ways that could potentially contribute to cancer development. For example, it has been shown that LINE-1 can cause DSBs via enzymatic activity of ORF2p and retrotransposition [136,137,138]. TE insertion can also result in altered genomic DNA secondary structures that can cause replication fork stalling and potential dissociation [139]. LINE-1 and ALU sequences have been shown to be involved in chromosomal deletions and rearrangements [140,141,142], and Chan et al. demonstrated that ncRNAs derived from α-satellite sequences can induce genomic instability through mitotic errors [143]. These α-satellite sequences were expressed via transfected expression vectors, but this could recapitulate the derepression of REs seen with loss of epigenetic silencing. Furthermore, a recent study from our lab showed that the derepression of REs led to replication stress and DNA damage [144]. It is conceivable that the induction of DNA damage by RE activation could induce DNA repair signaling mechanisms in these cells. Interestingly, it has been reported that activation of REs can also affect DNA repair pathways themselves. In breast cancers, de novo ALU insertions have been shown to disrupt the BRCA1/BRCA2 genes, which are involved in DNA repair [145,146]. Additionally, ALU sequences have been shown to interrupt mismatch repair (MMR) by altering the MLH1 and MLH2 genes [147,148]. In conclusion, these findings suggest that transcription and transposition of derepressed REs can jeopardize genome stability and induce DNA damage—things that can have a profound impact on cancer progression and evolution.

### 4.2. REs in Aging, Neurological Disorders, and Neurodevelopmental Diseases

#### 4.2.1. Role of REs in Aging

Aging is a major risk factor for a plethora of diseases, including neurological diseases described below. Aging is characterized by several hallmarks, and alteration of the epigenetic landscape is one of them [149]. Perhaps as both a result of and a reason for these epigenetic changes, RE activation has been shown to concomitantly increase with aging in several organisms, including humans. Studies conducted using drosophila as a model system showed that loss of heterochromatin during aging led to the derepression of REs [150,151]. Additional studies in drosophila showed that, with increasing age, repressive heterochromatin mark H3K9me3 and HP1 (heterochromatin protein 1) decreased, resulting in increased expression of REs [152]. In addition, another study showed that overexpression of proteins involved in the maintenance of heterochromatin mitigated the activation of REs [153]. Aged tissues from mice, humans, and non-human primates displayed increased expression of REs [108,154]. Another study in aged humans reported increased RE expression along with reduced DNA methylation at RE sequences [155]. Liu et al. also demonstrated that, unlike serum from younger humans, serum from aged humans induced senescence and inflammation when given to recipient cells. Importantly, this phenotype was rescued when the serum from the older individuals was treated with an anti-HERV-K envelope protein antibody (thus depleting the RVLPs from the serum). This suggests that HERV-K-derived RVLPs could also function as a biomarker for senescence and chronic inflammation associated with aging [108]. REs can also contribute to other significant hallmarks of aging. As mentioned above, derepressed REs can promote senescence, SASP, and sterile inflammation [107,108,155]. In addition, the shortening of telomeres (telomere attrition) is another hallmark of aging. Shortened and lengthened telomeres have been shown to decrease and increase lifespan, respectively, and reactivation of telomerase activity was shown to reverse age-associated tissue degeneration [149,156,157].

#### 4.2.2. Role of TEs in Neurological Diseases

Neurological diseases (NRDs) affect millions of people worldwide every year and cause a heavy economic burden on societies. NRDs can be broadly classified into two groups: neurodegenerative diseases and neurodevelopmental diseases. A central characteristic of NRDs is progressive neuronal loss resulting in depletion of normal brain and body functions. However, the etiology and molecular mechanisms involved in the onset of NRDs are poorly understood. Derepression of TEs represents one such molecular mechanism that has been linked with several NRDs. In this section, taking a few NRDs as examples, we explore the concepts behind the putative roles of TEs in NRD pathology. Increasing our understanding of the molecular mechanisms associated with the initiation and progression of NRDs could lead to the development of effective therapeutic interventions in the future.

##### Role of TEs in Neurodegenerative Diseases

Perturbed TE activity has been linked to various neurodegenerative disorders, and a subset are discussed in this section. Amyotrophic lateral sclerosis (ALS) is one of the better-studied examples exemplifying the relationship between abnormal expression of TEs and the progression of neurodegenerative diseases. ALS is characterized by the selective deterioration of motor neurons, eventually causing death. Several independent studies have shown evidence of increased reverse transcriptase activity in the serum of ALS patients. This has since been attributed to the presence of HERV-derived transcripts and proteins in serum, cerebrospinal fluid, and postmortem brain tissue of ALS patients [158]. In addition, the presence of protein aggregates was reported in ALS tissue samples, causing neuronal cell death and decreased cognitive abilities. These intraneuronal protein aggregates were shown to be formed by the RNA/DNA binding protein TAR DNA-binding protein 43 (TDP-43). The cytoplasmic aggregation of TDP-43 in neuronal cells made it disappear from the nucleus, leading to chromatin remodeling and the activation of TEs such as LINE-1 [159,160]. In agreement with these findings, another independent study that analyzed the transcriptomes of several ALS postmortem cortexes found that a subset of ALS patients with TDP-43 dysfunction had increased expression of TEs, especially LINE-1 and SVA elements [161]. As discussed in previous sections, abnormal TE expression can induce DNA damage and genomic instability, leading to inhibition of normal cellular functions and cell death. However, further studies are required in the context of ALS to mechanistically understand how their activation leads to the death of motor neurons.

Similarly, TEs have been implicated in Alzheimer’s disease (AD). AD is characterized by the loss of neuronal cells in the central nervous system (CNS), the presence of intracellular tangles of Tau protein, and accumulation of amyloid plaques. Several recent studies have shown elevated expression of TEs (i.e., HERVs, ALUs, LINE-1) in the brains of AD patients [162,163,164,165]. Furthermore, other mechanistic studies in mice, drosophila, and a human neuroblastoma cell line showed that overexpression of Tau protein led to global loss of heterochromatin, reduced the post-transcriptional silencing of TEs through the PIWI pathway, and activated TEs [162,166,167]. In addition, TDP-43 was also shown to regulate the expression of TEs via R-loops (RNA/DNA hybrids) and 5-hydroxymethylcytosine (5hmC) in the context of AD. That study indicated that cytoplasmic aggregation of TDP-43 in neuroblastoma cells leads to aberrant R-loop-5hmC crosstalk that could promote increased expression of TEs. However, how this crosstalk mechanistically drives TE expression remains unclear [168]. Furthermore, several recent studies have tried to explain how expression of TEs can cause neuronal cell death. Dembny et al. recently showed that RNA derived from the HERV-K envelope gene region can bind to murine toll-like receptor 7 (Tlr7) expressed in neurons and microglia, thereby causing neurodegeneration. In that study, the authors also reported a correlation between elevated RNA levels of HERV-K (HML-2) and TLR8 in AD brain samples, which implied that a HERV-K (HML-2)/TLR8 axis could have been driving the neurodegeneration process [169]. Another recent study showed that HERV-K-derived RNA/DNA hybrids can activate the cGAS-STING pathway in AD progenitors. While further exploration is needed, these findings suggest that activation of the cGAS-STING pathway by activated TEs can induce neuroinflammation—a hallmark of AD pathogenesis. That study also showed increased cleavage of caspase 3, a marker of apoptosis, by HERV-K-derived RNA/DNA hybrids in neurons [170]. This suggests that abnormal expression of TEs can also induce neuronal cell death in the context of AD—a finding that also warrants further exploration. Several possible mechanisms by which TEs can induce neuronal cell death and neurodegeneration are reviewed in [171].

Another neurodegenerative disease that has been associated with the activation of TEs is Parkinson’s disease (PD). PD is the second most common neurodegenerative disease, affecting millions of people worldwide. Notable clinical features of PD include degeneration of dopaminergic neurons in the substantia nigra leading to loss of dopamine, thereby causing resting tremors, bradykinesia, and rigidity in affected individuals [172,173]. It has been shown that LINE-1 expression is associated with PD [174,175]. Using a PD mouse model, it was shown that inhibition of LINE-1 through overexpression of the PIWI pathway protein Piwil1 reduced neuronal cell death [175]. In addition, increased expression of TEs was reported in human PD patient samples [176]. These studies indicate that TEs are associated with PD pathology, but further work is needed to increase our understanding of the mechanisms by which TEs could promote PD. Collectively, all the above-discussed studies indicate that when the activity of TEs becomes unchecked, it can lead to the induction of neuroinflammation and neuronal cell death, which are causative factors for neurodegenerative diseases.

##### Role of TEs in Neurodevelopmental Diseases

Studies have reported epigenetic alterations and activation of TEs in several neurodevelopmental diseases such as autism spectrum disorder, Rett syndrome (RTT), schizophrenia (SZ), and Aicardi–Goutières syndrome (AGS) [177,178,179,180]. TEs and their roles in some of these neurodevelopmental diseases are discussed below. RTT is a neurodevelopmental disorder with clinical features that include stereotypical hand movements, regression of spoken language, and gait abnormalities [181]. RTT is mostly caused by mutations in the X-linked methyl CpG binding protein 2 (MECP2) gene [182]. MECP2 encodes an epigenetic regulatory protein that binds methylated cytosines in CG and CA contexts and interacts with transcriptional co-repressor complexes. Muotri et al. showed that activation of LINE-1 elements was associated with disease phenotype in RTT and was modulated through MECP2 [180]. Schizophrenia is a neuropsychiatric disorder comprising cognitive, behavioral, and emotional disturbances, with the most common psychotic symptoms being hallucinations, disorganized thinking, and abnormal motor behavior. Increased LINE-1 insertions have been reported in schizophrenia patients [183,184]. In the case of AGS, which can also be considered an autoimmune disorder, defective RNAse enzymes are considered a major cause of the associated inflammation and interferon signaling [185]. RNAses are also implicated in the degradation of retrotransposon transcripts; thus, buildup of retrotransposon sequences are thought to be a driver of AGS inflammatory pathology through cytosolic nucleic acid sensing pathways [186]. Since TEs can stimulate the immune system through these sensing pathways, it is feasible that TE-induced neuroinflammation could lead to neurodevelopmental diseases such as RTT, SZ, and AGS. In summary, it is evident that aberrant activity of TEs is associated with various NRDs, and further information on this subject has been reviewed elsewhere [186,187]. To completely understand the role of TEs in the context of NRDs, several interesting questions remain unanswered: which TEs are activated in each neurological disease type and tissue context; whether these TEs are present in all patients with the disease or only a subset; and which silencing mechanisms are altered to enable TE activity.

## 5. Therapeutic Applications of RE Modulation

**Figure 3:**
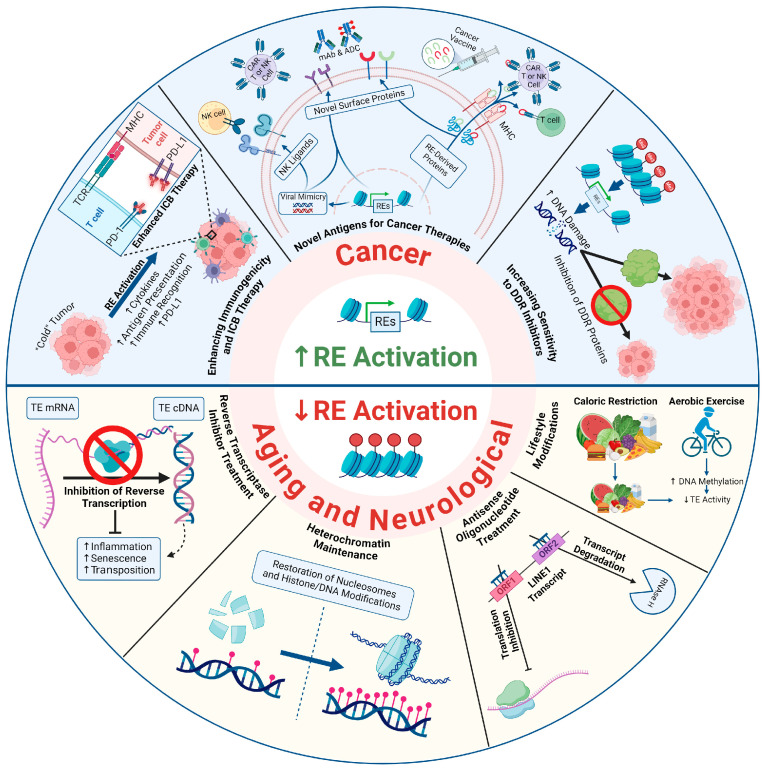
**Potential therapeutic applications of RE modulation.** This figure represents a non-exhaustive list of potential therapeutic avenues related to modulating RE expression. Information is presented in Section 5.1 and Section 5.2 of the text. Importantly, the top half of the figure represents potential cancer therapeutic strategies in which it is beneficial to induce RE activation. The bottom half represents aging and neurological diseases in which it is beneficial to repress RE activity. Created with BioRender.com (Accessed on 14 May 2024).

In this section, we discuss various potential strategies for harnessing RE modulation for the development of novel therapeutic strategies to treat human diseases, with a particular focus on combinatorial approaches for cancer treatment (Figure 3). 

### 5.1. Modulating RE Activity in Anti-Cancer Therapies

The past several years have seen tremendous advancements in the field of immune-oncology, including monoclonal antibodies, cancer vaccines, and chimeric antigen receptor (CAR)-T cells. Although these approaches look promising, they have unfortunately demonstrated limited success in clinical settings. Therefore, there is a clinical need to find novel effective strategies that can augment these immunotherapeutic approaches for cancer treatment. Because of the substantial knowledge that we now have regarding RE regulation and the associated antigens derived from them, there is growing interest among researchers in exploiting RE modulation for cancer treatment [69,188,189,190,191,192]. In this section, we discuss potential applications of RE modulation in augmenting therapeutic anti-cancer approaches.

#### 5.1.1. Activation of REs in Combination with Checkpoint Blockade Inhibitors

Induced expression of otherwise silent REs in cancer cells beyond a threshold level of tolerance elicits cellular viral defense mechanisms that include both innate and adaptive immune responses in a phenomenon termed viral mimicry (Figure 1). After two remarkable studies that initially led to the discovery of the viral mimicry concept were published [193,194], we and several other groups using different tumor models showed that the derepression of REs via various epigenetic inhibitory drugs led to the accumulation of cytoplasmic dsRNA and dsDNA, thereby activating viral mimicry responses [195,196]. In addition, our study showed that cytosolic dsDNA in cancer cells had markers indicating its derivation from genomic instability caused by activated REs. However, cytosolic dsDNA could also potentially accumulate in cells via reverse transcription of derepressed TE transcripts. Viral mimicry leads to the activation of antiviral and antitumor immune responses, including expression of ISGs, upregulation of MHC-I mediated antigen presentation, infiltration of cytotoxic immune cells (e.g., CD8^+^ T cells), and activation of immune checkpoint proteins such as PD-L1 (Figure 1 and Figure 3) [144]. These properties show that viral mimicry can stimulate antitumor immune responses in tumors that are otherwise unresponsive to immunotherapy (i.e., immunologically “cold” tumors). Cold tumors display an immunosuppressive tumor microenvironment (TME) that make them less responsive to immunotherapy. Their main characteristics include poor antigen availability, low tumor mutational burden, an absence of infiltrating cytotoxic immune cells, and the presence of immunosuppressive cells (e.g., M2 macrophages), among others. In contrast, immunologically “hot” tumors are immunostimulatory, with infiltration of CD8^+^ T and other cytotoxic immune cells, high tumor antigen availability, high tumor mutational burden, and anti-tumor M1 macrophages [197]. Using breast cancer as a model, our lab showed that the activation of REs by SUV39H1 inhibition synergized with anti-PD-1 immune checkpoint blockade (ICB) therapy in mice. SUV39H1 is an HMT, and its inhibition activated REs in cancer cells through loss of repressive H3K9me3 histone marks. That study also demonstrated increased infiltration of cytotoxic CD8^+^ and CD4^+^ T cells and NK cells in breast tumors [144]. Similar benefits of inducing viral mimicry in combination with ICB therapies have been observed through targeting several other enzymes, including EZH2, DNMTs, histone deacetylases (HDACs), MEK1/2 (kinases involved in the mitogen-activated protein kinase pathway), CDK4/6 (cyclin-dependent kinases), and DHX9 (RNA helicase) [198,199,200,201,202]. The potential clinical benefits of viral mimicry-inducing drugs with ICB therapies are being evaluated in several clinical trials [203].

#### 5.1.2. Antigens Derived from REs as Vaccination Targets

Targeting RE-derived tumor-associated antigens (TAAs) for vaccine development has great clinical potential because it can promote tumor regression by activating immune responses. Notably, several TAAs derived from REs that can potentially elicit antitumor immune responses were reviewed by Chen et al. [69]. It has been shown that prophylactic vaccination targeting antigens derived from HERVs can potentially elicit long-term immune responses in breast cancer [204]. In addition, several studies using preclinical animal models have suggested that therapeutic vaccination strategies could work in cancers where HERV-derived epitopes are processed and presented [205,206,207]. Importantly, when the safety of these vaccination strategies was investigated, no adverse side effects were reported [208]. Therefore, the evidence discussed in the above-mentioned studies indicates that TAAs derived from REs are well positioned to serve as important targets for designing vaccines against multiple cancers. However, there is a knowledge gap in this research area that hinders the development of cancer vaccines. For example, we know about only a few TAAs expressed in certain cancers. Future studies should focus on increasing the repertoire of potential RE-derived TAAs. This can be achieved by searching for novel TAAs or RE-derived peptides on MHC-I in cancer cells where REs are activated through gene knockout or treatment with epigenetic drugs [209]. Interestingly, there is also a possibility that a subset of RE-derived TAAs could be commonly expressed in multiple cancer types. If identified, such common TAAs could potentially aid in the design of a common vaccine capable of targeting several cancer types. Nevertheless, it is important to consider that TAAs derived from REs in cancer cells can also be expressed to some extent in normal cells. Therefore, further research is needed to identify RE-derived TAAs that are expressed only in cancer cells, to avoid off-target effects associated with many current standard-of-care immunotherapies and chemotherapies.

#### 5.1.3. REs and the Development of Cellular Immunotherapies

Adoptive cellular immunotherapy with CAR-T cells has emerged as an approach for cancer treatment. Since RE-derived peptides are a good source of TAAs, they can also be potentially used for designing CAR-T cell-based therapies. Krishnamurthy et al. showed that engineered CAR-T cells targeting the HERV-K envelope protein expressed in melanoma cells were able to reduce tumor burden in vivo. That study also showed that CAR-T cells were able to specifically kill tumor cells expressing HERV-K envelope on their surfaces [210]. Zhou et al. reported similar findings using CAR-T cells targeting HERV-K envelope protein in a breast cancer model [211]. In addition, HERV-K envelope protein expression has also been reported in pancreatic cancer cell lines and patient samples [212], though CAR-T-cell-mediated killing has not yet been tested. Shah et al. recently identified several chimeric, RE-derived proteins on the surfaces of cancer cells [191]. This newly identified surface repertoire can be used for engineering CAR-T-cell therapy against different cancers. Together, these studies indicate that TAAs derived from REs can potentially be used for the generation of CAR-T-cell-based therapies. However, it is important to note that CAR-T cells can cause severe toxic effects, such as cytokine release syndrome (CRS) and neurotoxicity [213]. Therefore, future studies should also consider this drawback when developing adoptive cell-based immunotherapies.

Recently, natural killer (NK) cells have emerged as potential candidates for developing novel cellular immunotherapies. NK cells are a major class of lymphocyte that function as a first line of defense against virus-infected and cancerous cells in the body. Like CAR-T cells, NK cells can also be engineered to express CARs that redirect the anti-tumor specificity on an antigen-dependent basis. However, compared with CAR-T cells, CAR-NK cells exhibit less toxicity [214]. Moreover, NK cells are allogeneic and not restricted by HLA expression. These properties suggest that CAR-NKs could represent a powerful strategy for treating human malignancies. The CAR for NK cells can be developed by utilizing various NK cell receptors expressed on the target tumor cell. For example, CAR-NK cells can be developed using natural killer group 2D (NKG2D)-CAR constructs, which specifically target and kill tumor cells expressing NKG2D ligands such as ULBPs. Therefore, NKG2D-CAR-NK cells can be used in combination with drugs that increase the surface expression of NK cell ligands on tumor cells. Recently, we showed that viral mimicry induced by SUV39H1 targeting increased the surface expression of NK cell ligand ULBP2 on breast cancer cells. Similarly, other viral-mimicry-inducing epigenetic drugs have also been shown to induce NK cell ligands (MICA/B and ULBP2) [144,215]. In addition, recent work from our lab showed that viral mimicry induced by SUV39H1 inhibition stimulated the cGAS-STING pathway, which Berger et al. showed can promote NK-cell-mediated tumor regression [216]. This is an emerging concept, but these studies provide strong rationale that combining CAR-NK cell therapy with viral-mimicry-inducing epigenetic therapies could be a powerful combination therapy for cancer. Furthermore, the derepression of REs can lead to presentation of new antigens on MHC-I, expression of RE-derived chimeric proteins on the cell surface, and increased expression of surface proteins activated by cellular stress or the viral mimicry process. Once identified, these antigens could also be potential candidates for the development of both CAR-T and CAR-NK cell therapies.

#### 5.1.4. REs as Targets for Monoclonal Antibody-Based Therapies

Monoclonal antibodies are considered important anticancer agents that enhance immune system functions to suppress cancer cell activity and eliminate cancer cells from the host. Monoclonal antibodies are produced by B cells, and they target a single epitope on their specific antigen. The idea of using monoclonal antibodies to target RE-derived TAAs emerged from a study conducted by Wang-Johanning et al. As proof of concept, the authors demonstrated that monoclonal antibodies targeting the HERV-K envelope protein reduced the proliferation of breast cancer cells in vitro and significantly reduced tumor growth in mice [217]. In line with this finding, another recent study showed that HERV-K-envelope-targeting antibodies mediated potent anti-tumor effects in lung cancer [218]. Together, these studies provide evidence that monoclonal antibodies targeting RE-derived TAAs could potentially be used as anti-cancer therapies.

Antibody drug conjugates (ADCs) are another class of therapeutics which have recently emerged as promising anticancer agents [219]. ADCs consist of a monoclonal antibody (mAb) covalently attached to a cytotoxic drug via a chemical linker. The ideal antigen for ADCs should be highly expressed on cancer cells with minimal to no expression on normal cells. Therefore, antigens like the HERV-K envelope protein, which is expressed mainly on cancer cells, could be a promising antigen for ADCs. However, one of the major challenges of antibody therapies is low target expression on cancer cells. One way to potentially overcome this issue could be through activating REs selectively in cancer cells, leading to increased expression of TAAs that can be targeted by novel antibody therapies. Thus, future studies should focus on identifying novel RE-derived TAAs. The study conducted by Shah et al. discussed above is one such example [191].

#### 5.1.5. Challenges to Be Addressed for RE-Based Immunotherapeutic Applications

Clinically, there are several challenges that still need to be addressed for the successful development of RE-based cancer therapies. While not necessarily an exhaustive list, a few pertinent questions include (1) whether RE derepression generates antigens in a tumor- and tissue-specific manner, (2) whether this strategy will be effective in tumors that mutate or downregulate genes associated with antigen presentation [220] or interferon signaling pathways [221,222], and (3) whether efficient targets and drugs can be identified that selectively activate REs in cancer cells but not normal cells. In addition, most of the studies discussed in the previous sections demonstrated the potential benefits of RE activation in either human cell lines or murine models. Where possible, future work should explore RE activity and modulation in patient-derived cells to increase the potential clinical translation of any findings.

#### 5.1.6. RE Activation and DNA Damage Response Inhibitors

Recent studies have begun to elucidate the interplay between RE activation and DNA damage. When DNA damage occurs in cells, DNA damage response (DDR) signaling mechanisms are activated that function to repair the damaged DNA. Therefore, targeting these DDR processes could induce excessive DNA damage and eventual cell death. Several studies have demonstrated that activation of LINE-1 causes genomic instability and DNA damage in cells [136,138,223]. REs have also been shown to form RNA–DNA hybrids and induce DSBs through replication fork collapse [224]. Work in our lab showed that derepression of REs can cause DNA damage and genomic instability, as evidenced by increased levels of DNA damage markers and activated DDR proteins [144]. Furthermore, Ardeljan et al. showed that LINE-1-expressing cells are dependent on DNA repair proteins such as BRCA1 and FANCD2 for growth [225]. Based on these findings, it is conceivable that simultaneously activating REs and inhibiting DDR pathways could induce synthetic lethality, ultimately leading to cancer cell death. Future studies should investigate, in a cancer-specific context, which DNA damage response signaling pathways are activated in response to RE-induced DNA damage and whether these can be therapeutically targeted for clinical benefit.

### 5.2. Targeting TEs in Aging and Neurological Disorders

In contrast to cancer, where the goal is to induce cancer cell death through the activation of REs and concomitant antiviral immune responses and genomic instability, the aim in aging and neurological disorders is the opposite: to inhibit aberrant TE activity to spare the afflicted tissues. Aberrant TE expression can be mitigated through several potential approaches (Figure 3). One of these involves using nucleoside reverse transcriptase inhibitors (NRTIs). Several studies have shown alleviation of age-associated inflammation and senescent phenotypes upon treatment with NRTIs [107,108,154] and there are currently several clinical trials for neurological disorders exploring NRTIs alone or in combination with anti-HERV-K integrase inhibitors that have been reviewed elsewhere [187]. Other therapeutic avenues that remain more theoretical at this point relate to mechanisms that promote heterochromatin stability and maintenance. For example, there have been reports of global histone loss associated with aging, and it is not farfetched to assume that this would allow increased access to many genomic regions (i.e., TE sequences). Along these lines, overexpression of histones 3/4 and deletion of genes involved in the transcriptional repression and degradation of histones provided increased lifespan [226,227,228]. Furthermore, DNMT1 expression and DNA methylation have been shown to generally decrease with age [229,230], suggesting this could be another mechanism by which TEs are aberrantly activated with aging. Thus, drugs that maintain DNMT1 levels (or perhaps inhibit “erasers” of DNA methylation) could be potential mechanisms for alleviating neurological and aging-associated pathologies. Another approach to target aberrant TE expression in these contexts is with antisense oligonucleotides (ASOs). Most of the following information on ASOs is from Rinaldi and Wood’s comprehensive review on the subject [231]. ASOs are oligonucleotide DNA sequences complementary to a portion of a transcript targeted for degradation or protein downregulation. In this context, ASOs could be designed to target various TE transcripts. For example, ASOs can cause degradation of the transcript through recognition of RNA–DNA hybrids by RNAseH1. Additionally, ASO binding can cause steric hindrance of the translation machinery, and ASOs designed against the intron/exon junctions of a transcript can alter splicing, resulting in defective protein products. Notably, ASOs can be designed to have several types of modified backbones to increase systemic stability and prevent nuclease degradation prior to their therapeutic effects. While the target selectivity conferred by ASOs is promising, other issues such as finding the proper systemic delivery systems, potential carriers, and backbone structures that promote the most efficient cellular uptake are still being fine-tuned. Importantly, a few ASO therapies are already used in humans, with several more trials ongoing [231]. In the context of decreasing TE activation, systemic introduction of LINE-1-targeting ASOs remedied several age-associated phenotypes in a mouse model of an early-aging disease [97]. Finally, aside from drug-based approaches, aerobic exercise was shown to reduce age-associated expression of TE transcripts and increase DNA methylation at TE sites [155]. Additionally, caloric restriction is another potential method that has been shown to increase lifespan and decrease TE expression for the alleviation of aging and neurological disease phenotypes [232,233].

## 6. Concluding Remarks and Future Perspectives

The numerous studies and concepts discussed here highlight that REs are important players in the (dys)regulation and evolution of their host’s genome (and thus, evolution of the host organism). Furthermore, their roles in the conditions reviewed here (and others) suggest that targeting REs may address aspects of disease pathologies that were previously difficult to ameliorate. However, there are still important knowledge gaps which need to be filled. For example, in cancer, identifying ways to efficiently derepress REs selectively in cancerous cells will facilitate the development of novel therapies while limiting potential systemic inflammatory side effects. In aging and neurological conditions, further characterization of the mechanisms by which TEs become aberrantly expressed in different disease states is paramount for the design of novel treatment options. Despite these challenges, the future looks bright regarding RE biology, for several reasons.

First, advances in sequencing technologies are making it easier to sequence repetitive DNA regions and elucidate the epigenetic marks associated with them. The implication of REs in inflammatory processes also represents a promising new way to address conditions where chronic inflammation is a hallmark (whether through direct RE targeting or modulation of sensing pathways [74]). Additionally, as systemic gene therapy technologies advance, it is not so difficult to speculate that deleterious TE activities could be systemically silenced to reduce their harmful effects in aging and neurological diseases. Lastly, the “transposable element toolbox”, where TEs are used as vectors for genome editing, is continuing to expand. For example, a 2024 study identified 40 new transposons capable of integration into the human genome. One of these newly identified transposons was used to generate CAR-T cells that outperformed those generated with other typical vectors in the killing of blood cancer cells [234]. In summary, one man’s junk (DNA) is another man’s treasure, and the ways that REs have influenced the human genome and continue to do so could be leveraged to advance the development of therapeutics that target a variety of human diseases.

## Supplementary Materials

The following supporting information can be downloaded at: https://www.mdpi.com/article/10.3390/biom14101250/s1, Figure S1: Classification of tandem and interspersed REs as described in the text. Green boxes represent transposable elements that are still capable of mobilization in the human genome. Created with BioRender.com (accessed on 14 May 2024).
