# Supplementary material for: Evolution of Repetitive Elements, Their Roles in Homeostasis and Human Disease, and Potential Therapeutic Applications
Source: Biomolecules. 2024 Oct 2;14(10):1250. doi: 10.3390/biom14101250 (PMC11506328; doi:10.3390/biom14101250)
Supplement: Supplementary file 1 [file biomolecules-14-01250-s001.zip › biomolecules-3190800-supplementary.pdf]

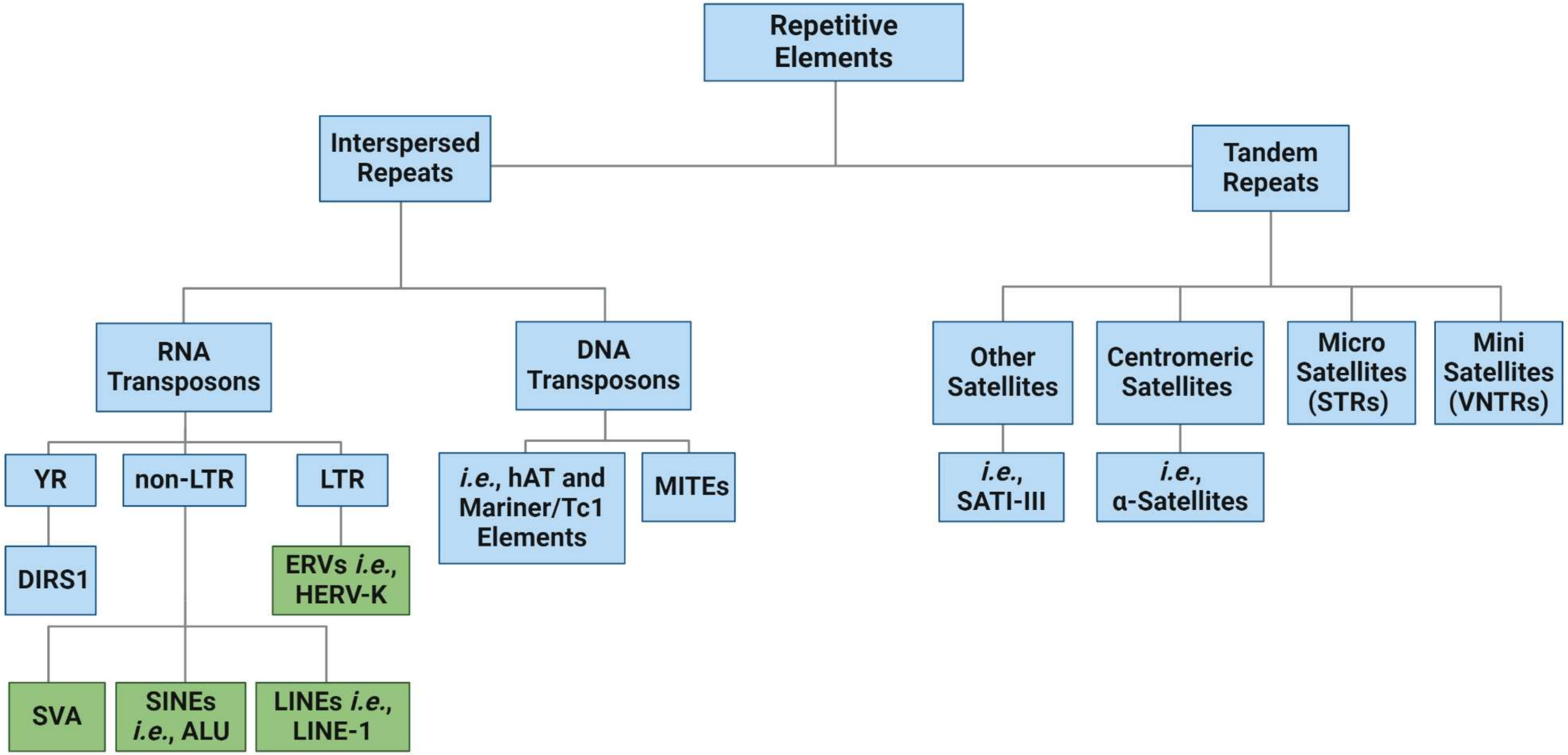

Supplemental Figure S1: Classification of tandem and interspersed repetitive elements as described in the text. Green boxes represent transposable elements that are still capable of mobilization in the human genome. Created with BioRender.com
